# Tempering of exhausted T cells to comprehend their adaptive response for suitable clinical translation

**DOI:** 10.1038/s41423-023-01031-y

**Published:** 2023-05-09

**Authors:** Amit Sharma, Ingo G. H. Schmidt-Wolf

**Affiliations:** Department of Integrated Oncology, Center for Integrated Oncology (CIO) Bonn, University Hospital Bonn, Bonn, Germany

**Keywords:** Cancer, Cancer microenvironment

Although cancer immunotherapy has taken center stage, exhaustion of T cells is undeniably one of the major problems preventing it from being fully successful [1]. Certainly, reversal of T-cell exhaustion or dysfunction is of great importance for developing new strategies and restoring immune function. Nevertheless, counterarguments about the adverse effect of reversing T-cell exhaustion (leading to overactivation of T cells and cytotoxicity) and even their irreversible properties have been quite common in scientific debates. With more to be discovered about the reasons for T-cell failure, a better understanding of the molecular factors/determinants [2, 3] that shape/remodel “heterogeneous” dysfunctional T cells in cancer makes clear sense. Indeed, two recent publications in *Nature immunology* have described the intriguing perspective of T-cell exhaustion [4, 5]. Of interest, Vignali et al. focused on the hypoxia-driven CD39-dependent suppressor function of exhausted T cells, whereas Saadey et al. emphasized restoring the epigenetic balance of TGFβ1/BMP signaling in exhausted T cells to unlock responsiveness to immune checkpoint blockade therapy.

In a very comprehensive analysis, Vignali and colleagues showed that CD4 + Treg cells and CD8 + terminally exhausted T cells (tTex) share some overlap in transcriptional programming in the tumor microenvironment (TME) [2]. This may in fact be supportive of exhausted CD8 + T cells possessing a suppressive phenotype upon terminal differentiation. Of note, the authors showed that tTex cells exert their inhibitory function via CD39 and that its deletion in CD8 + tTex cells enhances the immunotherapy response. Their study also highlighted that tumor hypoxia enforces the suppressor functions of tTex cells through increased CD39 expression, thereby generating adenosine and consequently limiting therapeutic efficacy. The authors defined tTex cells not only as a hypofunctional state, but one that may conditionally have strong anti-functional features that suppress local T-cell immunity. Apparently, hypoxia, as the predominant feature of the TME, is itself immunosuppressive. It has been well established that tumor-associated hypoxia can affect the function of many immune cells, and as recently described, exhausted T cells are no different [6]. Therefore, Vignali et al. clearly provided interesting insight into the mitigating role of hypoxia in exhausted T cells. Independently, Saadey and colleagues explored the signals that regulate epigenetic programming in dysfunctional T cells and investigated the role of microenvironmental signals in the epigenetic regulation of chronically stimulated CD8 + T cells at the posteffective stage [3]. The authors developed an in vitro model system for stable human T-cell dysfunction and showed that chronic TGFβ1 signaling in posteffector CD8 + T cells accelerates their terminal dysfunction through stable epigenetic changes. Interestingly, an exciting new and decisive role for TGFβ1/BMP signaling as key factors in epigenetic remodeling and cell fate commitment of dysfunctional CD8 + T cells was defined by this study. In particular, the key message about rebalancing TGFβ1/BMP signals as a new approach to unleash dysfunctional CD8 + T cells and enhance T-cell immunotherapies will undoubtedly open new avenues for further research in this field. It is also worth acknowledging that the authors examined closely but found no evidence of bystander activation of CD8 + T cells, thus providing a rationale to further investigate the extent to which epigenetic manipulation can combat this in the clinical setting.

Exhausted T cells have long been considered dysfunctional with defined expression of multiple coinhibitory receptors (e.g., PD-1, TIM-3, LAG-3), leading to failed antitumor responses. The aforementioned studies provide an additional layer of scientific evidence regarding the relative contribution of hypoxia and possible molecular regulation of T-cell exhaustion, with an emphasis on epigenetic mechanisms. More studies in this direction can certainly help to improve our understanding of T-cell exhaustion and co-orchestrate therapies, e.g., chimeric antigen receptor (CAR)-modified T cells (CAR-T) [7, 8] and T-cell receptor (TCR)-engineered T cells (TCR-T) [9], which rely entirely on the functional perspective of T cells. It is worth noting that in the race of adoptive cell therapies, cytokine-induced killer (CIK) cell immunotherapy, which contains a significant proportion of T-cell subtypes, may also be of major interest [10].
